# An improved advanced fragment analysis-based classification and risk stratification of pediatric acute lymphoblastic leukemia

**DOI:** 10.1186/s12935-019-0825-y

**Published:** 2019-04-25

**Authors:** Yanran Sun, Qiaosheng Zhang, Guoshuang Feng, Zhen Chen, Chao Gao, Shuguang Liu, Ruidong Zhang, Han Zhang, Xueling Zheng, Wenyu Gong, Yadong Wang, Yong Wu, Jie Li, Huyong Zheng

**Affiliations:** 1 Beijing Key Laboratory of Pediatric Hematology Oncology, National Key Discipline of Pediatrics (Capital Medical University), Key Laboratory of Major Diseases in Children, Ministry of Education, Hematology Oncology Center, Beijing Children’s Hospital, Capital Medical University, National Center for Children’s Health, 56 Nanlishi Road, Beijing, 100045 China; 2 School of Computer Science and Technology, Harbin Institute of Technology, 92 West Dazhi Street, Nan Gang District, Harbin, 150001 Heilongjiang China; 3 Center for Clinical Epidemiology & Evidence-based Medicine, Beijing Children’s Hospital Medical, Capital Medical University, National Center for Children’s Health, 56 Nanlishi Road, Beijing, 100045 China; 4 Ningbo Health Gene Technologies Ltd., Ningbo, 315800 Zhejiang China; 5 Present Address: Institute of Medical Biology, Chinese Academy of Medicine Sciences and Peking Union Medical College, 935 Jiaoling Road, Kunming, 650031 Yunnan China

**Keywords:** Acute lymphoblastic leukemia, Pediatric, Classification, Risk stratification, Prognosis

## Abstract

**Background:**

Acute lymphoblastic leukemia (ALL) contains cytogenetically distinct subtypes that respond differently to cytotoxic drugs. Therefore, subtype classification is important and indispensable in ALL diagnosis. In our previous study, we identified some marker genes in childhood ALL by means of microarray technology and, furthermore, detected the relative expression levels of 57 marker genes and built a comparatively convenient and cost-effective classifier with a prediction accuracy as high as 94% based on the advanced fragment analysis (AFA) technique.

**Methods:**

A more convenient improved AFA (iAFA) technique with one-step multiplex RT-PCR and an anti-contamination system was developed to detect 57 marker genes for ALL.

**Results:**

The iAFA assay is much easier and more convenient to perform than the previous AFA assay and has a prediction accuracy of 95.29% in ALL subtypes. The anti-contamination system could effectively prevent the occurrence of lab DNA contamination. We also showed that marker gene expression profiles in pediatric ALL revealed 2 subgroups with different outcomes. Most ALL patients (95.8%) had a good-risk genetic profile, and only 4.2% of ALL patients had a poor-risk genetic profile, which predicted an event-free survival (EFS) of 93.6 ± 1.3% vs 18.8 ± 9.8% at 5 years, respectively (*P* < 0.001).

**Conclusions:**

Compared to the previous AFA assay, the iAFA technique is more functional, time-saving and labor-saving. It could be a valuable clinical tool for the classification and risk stratification of pediatric ALL patients.

**Electronic supplementary material:**

The online version of this article (10.1186/s12935-019-0825-y) contains supplementary material, which is available to authorized users.

## Background

Genomic profiling has revolutionized our understanding of cancer and refined the classification of patients into clinically relevant subgroups [1]. This is exemplified in how risk classification and corresponding chemotherapy have improved the outcome for patients with pediatric acute lymphoblastic leukemia (ALL). Refinements in treatment protocols rely on accurate classification, and improvements in supportive care for ALL have led to a cure rate approaching 90% in recent years. However, certain high-risk pediatric ALL subgroups remain relatively intractable to treatment, and many patients who relapse face a similarly dismal outcome. Moreover, survivors who are low-risk patients suffer long-term sequelae from their intensive treatment throughout their lives. Therefore, reliable classification and stringent risk assessment have become extremely important issues to improve the survival of patients at high risk and decrease the long-term treatment-related side effects in standard-risk patients [2–5].

Microarray technology was exploited for the classification of leukemia for the first time in 1999 [6]. Since then, a substantial series of studies has revealed either novel subtypes or biomarkers for pediatric ALL based on high-throughput technologies [7–12]. In a previous study, we constructed a classifier of some marker genes that applies to a single independent patient sample and can consistently retain high accuracy with microarray technology [13]. Then, we adopted the advanced fragment analysis (AFA) technique, which integrates multiplex RT-PCR and capillary electrophoresis, to detect 57 marker genes effectively and economically. The AFA-based classifier also demonstrated high accuracy and was more rapid and inexpensive than a previous microarray-based classifier [14].

In this study, the efficiency and stability of the AFA technique was further improved with one-step multiplex RT-PCR and an anti-contamination system. In addition, the prognostic significance of marker genes in pediatric ALL was also evaluated.

## Materials and methods

### Patients and treatment protocol

A total of 219 newly diagnosed pediatric ALL patients, aged 5 months to 14 years (median, 4 years), were enrolled in the Hematology Oncology Center at the Beijing Children’s Hospital of Capital Medical University between May 2011 and August 2016. The 160 samples of our previous study between August 2007 and January 2014 were analyzed together in this study [14]. The clinical characteristics of all patients are described in detail in Additional file 1: Table S1. All patients were diagnosed with ALL using a combination of morphology, immunology, cytogenetics and molecular biology (MICM) classification. They were stratified and treated according to the China Children’s Leukemia Group (CCLG)-ALL 2008 protocol as previously described [15]. Minimal residual disease (MRD) was evaluated at the end of the induction of remission (day 33) and before consolidation (day 78). This method has been reported previously in detail [15, 16]. We defined MRD positive as ≥ 10^−2^ at day 33 and ≥ 10^−3^ at day 78 in this study. Due to induction failure or abandoning therapy, only 340 and 327 cases were available for MRD detection on the 33rd and the 78th days, respectively. Informed consent was obtained from all parents or legal guardians. The study was designed in accordance with the Declaration of Helsinki and was approved by the Beijing Children’s Hospital ethics committee prior to its initiation.

### Cell sample collection, RNA extraction, and primer design

Bone marrow (BM) samples were collected at the time of initial diagnosis (ID) into a tube with anticoagulant ethylenediaminetetraacetic acid. The inclusion criteria were sufficient BM cells for total RNA extraction and ≥ 50% blast cells in the BM samples. Mononuclear cells were isolated by Ficoll gradient centrifugation (MD Pacific, Tianjin, China; density: 1.077 g/ml) and cryopreserved in a − 80 °C freezer for subsequent experiments. Total RNA from the BM samples was extracted within 2 weeks using TRIzol reagent (Invitrogen, Paisley, UK) according to the manufacturer’s instructions. The concentration and quality of RNA were determined by absorbance measurements at 260 nm and 280 nm.

A previous study demonstrated that the primers we designed can effectively amplify all the marker genes. In addition to *B2M*, *PSMC4* and *GUSB* used as the endogenous reference genes for normalization and calculation of fold change and 2 internal reference controls, KanR and pcDNA3.1(+) serve as quality controls for the RT-PCR reaction [14]. In this study, the primer sequences of 57 marker genes and the concentration of each primer is presented in Additional file 1: Table S2.

### iAFA-based multiplex assay

We utilized a one-step multiplex RT-PCR assay and an anti-contamination system to improve the AFA assay. Uracil-DNA glycosylase (UDG) was used in the reaction system to digest the carry-over amplicons and prevent contamination. For each reaction, 50 ng/µl RNA from each sample was amplified directly with the following reaction system: 2 µl of RNA sample (50 ng/µl), 3 µl of RT-PCR primer mix, 2.5 µl of 5 × RT-PCR buffer (100 mM Tris–HCl at pH 8.3, 500 mM KCl, 25 mM MgCl_2_), 1 µl of KanR (0.25 ng/µl), 0.5 µl of RT-PCR Enzyme Mix [reverse transcriptase (10 units/µl), hot-start DNA polymerase (2.5 units/µl), UDG enzyme (0.5 units/µl)], and 1 µl of CM02; CM02 contains the fluorescence-labeled universal forward primer (12 µM), unlabeled universal reverse primer (12 µM), and dU-NTP [dATP, dCTP, dGTP (3.5 mM), dTTP, dUTP (1.75 mM)]. All reagents in the reaction were provided by Ningbo Health Gene Technologies Ltd. (Ningbo, China).

One-step multiplex RT-PCR reactions were then performed using a Veriti^®^ Thermal Cycler (Applied Biosystems, USA) as follows: 25 °C for 5 min, 50 °C for 30 min, 95 °C for 15 min, 35 cycles of 94 °C for 30 s, 60 °C for 30 s and 72 °C for 1 min, followed by 72 °C for 1 min of additional extension and holding at 4 °C.

An aliquot (1 µl) of the amplification product for each set was prepared for capillary electrophoresis by adding 31.8 µl of CEQ Sample Loading Solution (AB SCIEX, USA) and 0.2 µl of CEQ DNA Size Standard 400 (AB SCIEX, USA) in a 96-well CEQ electrophoresis sample plate (AB SCIEX, USA). The “Frag-3” separation method of the Beckman Coulter GeXP Genetic Analysis system (Beckman Coulter, USA) was also used to analyze amplification products. The relative quantification of each gene in a sample was determined using a standard curve as in a previous study [14].

### Data analysis

The AFA data for 160 children with ALL was published in our previous study [14]. The iAFA assay dataset consisted of 219 patients with 7 main subtypes: 12 patients with *E2A*-*PBX1*, 56 with *TEL*-*AML1*, 9 with *BCR*-*ABL1*, 71 hyperdiploid, 16 T-ALL, and 5 with *MLL* rearrangement. The remaining 50 B-ALL cases had not yet been detected for any fusion gene or chromosomal abnormality and were classified as “Others” for the time being.

Some missing values in the dataset were labeled as zero according to the principle of the iAFA assays that genes with very low expression did not exhibit signals. We also utilized the rank values instead of the true signal for each gene to construct the classifier. A decision tree (C5.0) classifier with optimal parameters was adopted to classify the seven subtypes of samples. C5.0 is implemented with “C50”, which is one of the most popular R packages for machine learning (http://www.r-project.org/).

### Statistical analysis

In the study, March 28, 2018, was used as the end of the collection of the patients’ treatment outcomes. Event-free survival (EFS) was defined as time to ALL relapse, second malignant neoplasms (SMNs) or death, with censoring at last contact.

Stepwise logistic regression was used to select marker genes that can predict the prognosis of ALL patients in this study. First, several candidate models were selected according to the corrected Akaike’s information criterion (AICc) and Bayesian information criterion (BIC) values. The lower the AICc and BIC values, the better the model. Then, fivefold cross-validation was used to determine the optimal model, and the model with the lowest misclassification rate in the validation set was considered the final prediction model. Finally, the patients were classified into two groups, the good-risk (GR) group and the poor-risk (PR) group, by the prediction model. The Kaplan–Meier method and log-rank test were used to estimate and compare the survival curves of the two groups. Comparisons between the clinical characteristics, early treatment responses and prognoses of the two groups were performed using the Chi square test or Fisher’s exact test, where appropriate. A *P* value of less than or equal to 0.05 was considered significant. All analyses were performed using SAS 9.4 and SPSS 16.0 for Microsoft Windows software.

## Results

### iAFA assay optimization for pediatric ALL subtypes

The iAFA assay was carried out using our previous method, and the 57 marker genes were randomly divided into 3 panels, with each panel containing 18 to 20 genes [14]. The concentration of each primer was optimized using our previous method, and *NEDD4* (in panel 3) did not reach moderate height, although we had raised its primer concentration. Therefore, 56 marker genes were used for subsequent assays.

**Fig. 1:**
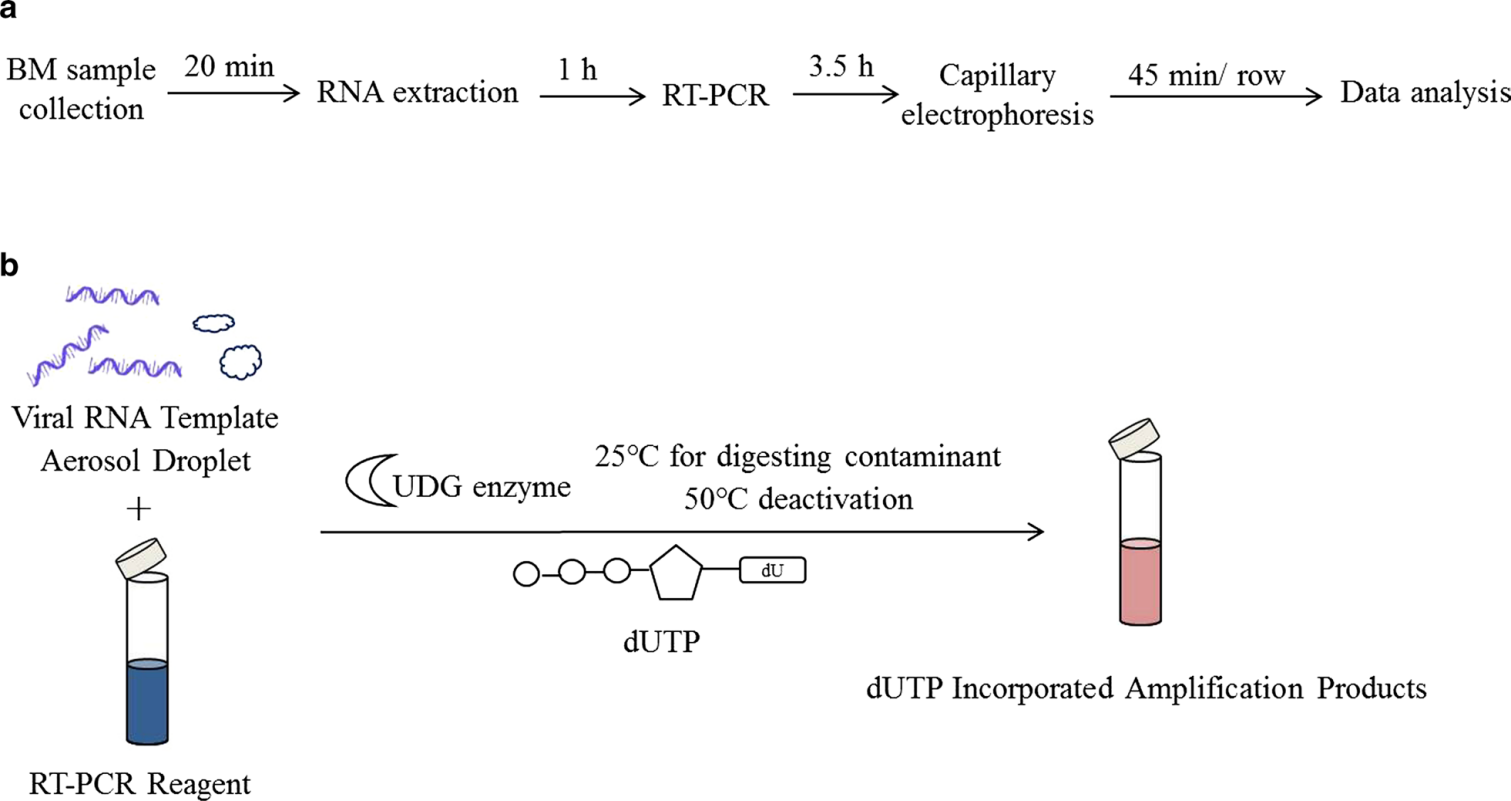
Schematic outline of the iAFA multiplex assay. **a** Schematic of the iAFA multiplex assay protocol, along with the approximate time required for each step. The assay integrated one-step multiplex RT-PCR and capillary electrophoresis to produce a time-saving and labor-saving method. **b** The UDG enzyme-based elimination of carryover contaminants by specifically cutting the 5′ side of the dUTP-incorporated amplicon DNA while having no effect on RNA templates. During the multiplex RT-PCR reaction, the possible contaminants were degraded into small fragments, and the UDG enzyme was inactivated at approximately 50 °C, ensuring that only the RNA template was amplified

In the iAFA assay, a UDG enzyme-based anti-contamination system was implemented. Compared to the previous assay, the iAFA assay was also supplemented with dUTP, which can incorporate dUTP into all RT-PCR amplicons after amplifying the target RNA sequence under certain conditions (“Materials and methods”). Such processing was the foundation of eliminating carryover contamination. To digest the dUTP-incorporated RT-PCR products, UDG treatment was performed before the reaction at 25 °C, whereas the template RNA, which does not contain dUTP, could not be digested under this step. In addition, the UDG enzyme is heat-sensitive and fully deactivates in the next step at 50 °C. Therefore, it did not affect the newly amplified RT-PCR products. The schematic diagram of the iAFA multiplex assay is illustrated in detail (Fig. 1). The UDG enzyme not only eliminated the carryover contamination dUTP-incorporated amplification products but failed to influence the amplification of marker genes. The stability and efficiency of amplification improved significantly.

**Fig. 2:**
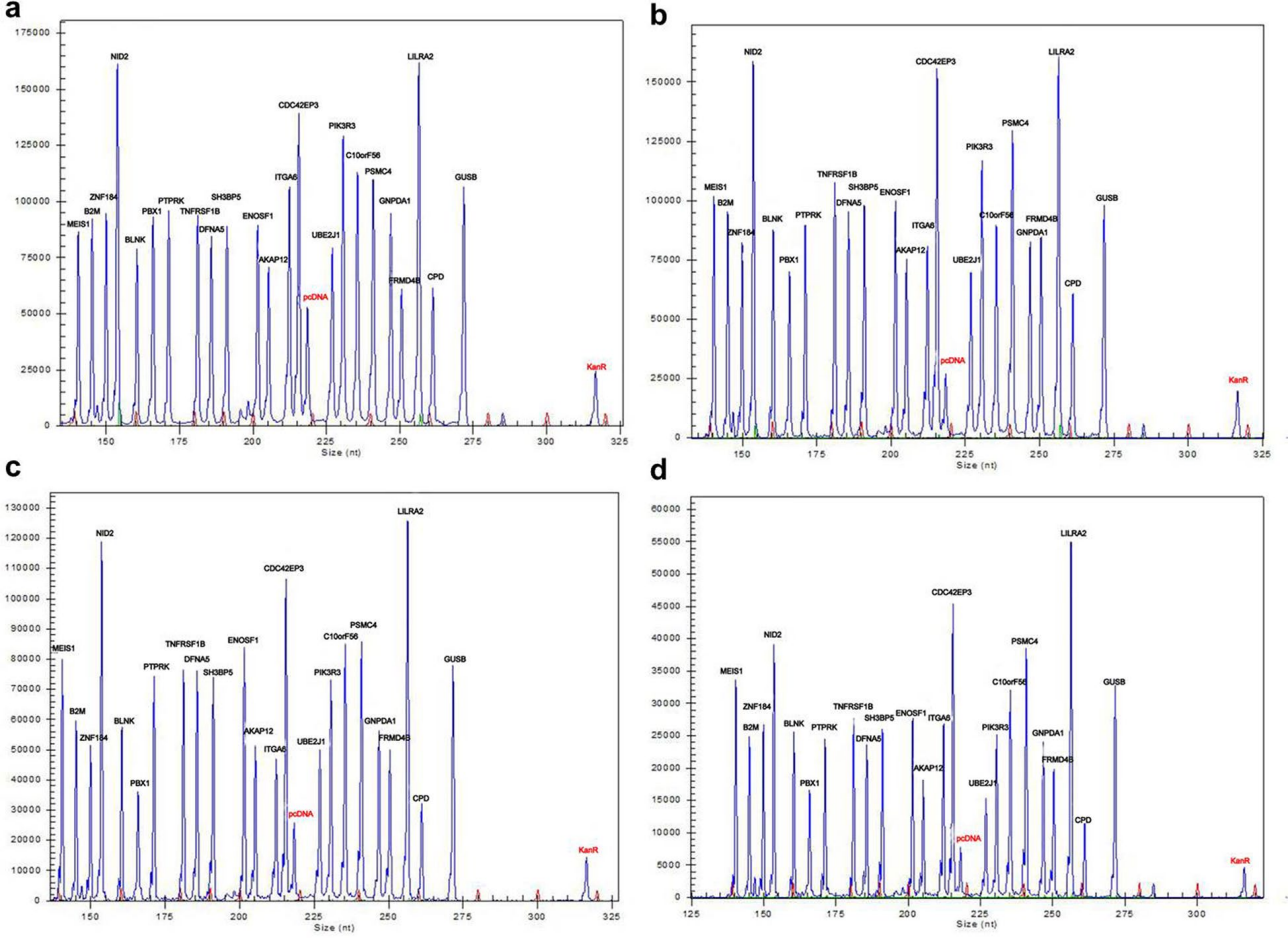
Different concentrations of dUTP had similar expression levels of marker genes in pediatric ALL. There was no significant disparity in the efficiency of amplification among the three panels. Hence, we chose only Panel 2 to determine the concentration of dUTP. **a**–**d** represent dUTP:dTTP = 0:3; 1:3; 2:3 and 3:3, respectively. **a** 3.5 mM dTTP; **b** 0.875 mM dUTP and 2.625 mM dTTP; **c** 1.4 mM dUTP and 2.1 mM dTTP; **d** 1.75 mM dUTP and 1.75 mM dTTP

Because the amplification efficiency of dUTP is slightly lower than that of dTTP in the reaction, we searched for the appropriate concentration of dUTP to ensure its amplification efficiency and anti-contamination effects. A gradient test of dUTP: dTTP (0:3; 1:3; 2:3; 3:3) was implemented, as shown in Fig. 2. There were no significant differences among the four gradient concentrations of dUTP. The results demonstrated that different doses of dUTP had little influence on the efficiency of amplification in this reaction when the space difference of the instrument was excluded. Consequently, dUTP:dTTP = 3:3 was selected for the subsequent assays.

**Fig. 3:**
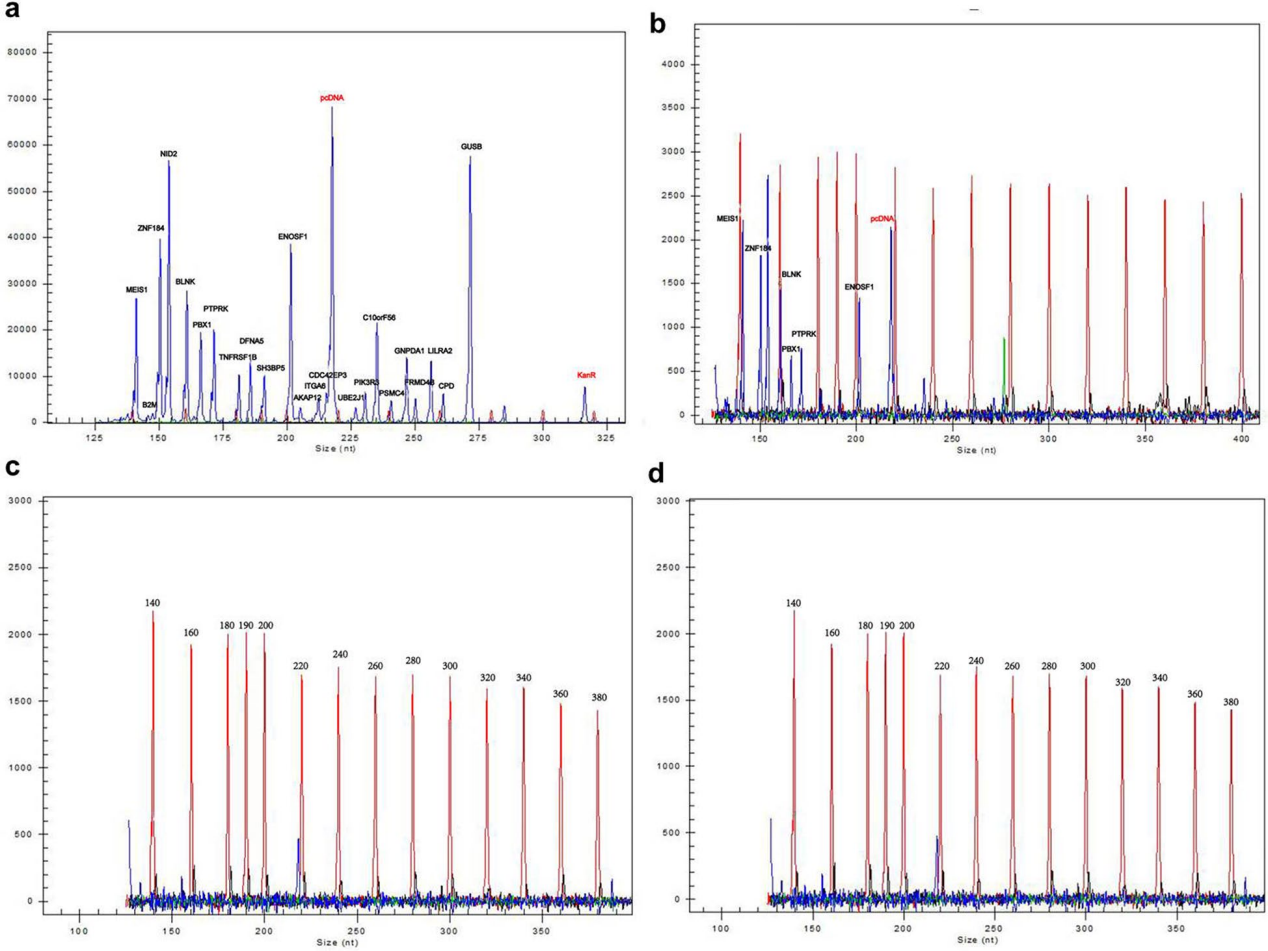
Decontamination ability of different doses of the UDG enzyme. The total dose of the UDG enzyme is 0 U, 0.125 U, 0.25 U and 0.375 U in **a**–**d**, respectively

The dose of the UDG enzyme also needed to be determined. In PCR, aerosol droplets are the most common and serious contamination source. We selected the dUTP-incorporated amplification products, which were diluted 10^7^-fold, instead of the RNA template in the 10 µl reaction system as simulated carryover contamination. Meanwhile, the decontamination ability of the UDG enzyme (0 U, 0.125 U, 0.25 U and 0.375 U UDG enzyme) was evaluated (Fig. 3). Our results indicate that 0.25 U UDG enzyme was sufficient to eliminate dUTP-incorporated carryover contamination in the reaction system (Fig. 3c).

### Development and independent validation of the classifier with iAFA samples

The C5.0 machine-learning method was used to train a classification model. We did not yet know whether the classifier was robust across different assays, so we first constructed the C5.0 model with tuning parameters based on our previously published 160 AFA data [14] and validated it with 219 independent iAFA samples. We investigated the classification performance through three measures: accuracy, sensitivity and specificity. The results of the 219 validated samples are shown in Additional file 1: Table S3. This classifier had an overall accuracy of 91.13%, indicating that it has strong robustness in different assays.

Then, the 219 iAFA samples were further divided into two groups using stratified random sampling: 111 cases were used as the training set, and the remaining 108 cases were used as the testing set. The analytical process was as follows: (i) the procedure was repeated for 10 runs, and the accuracy, sensitivity and specificity were computed for each run; and (ii) the average accuracy, sensitivity and specificity over the runs were computed, as presented in Table 1.

**Table 1:** Prediction results for the independent 108-case testing set of iAFA samples

Subtype	TP	FP	TN	FN	Accuracy (%)	Sensitivity (%)	Specificity (%)
*BCR*-*ABL1*	1.7	2.3	101.7	2.3	95.74	42.50	97.79
*E2A*-*PBX1*	6	0.3	101.7	0	99.72	100.00	99.71
Hyperdiploid	30.1	7.5	65.5	4.9	88.52	86.00	89.73
*MLL* rearrangement	1.3	0.3	105.7	0.7	99.07	65.00	99.72
Others	15.8	4.7	78.3	9.2	87.13	63.20	94.34
T-ALL	7.9	1.1	98.9	0.1	98.89	98.75	98.90
*TEL*-*AML1*	27.4	1.6	78.4	0.6	97.96	91.33	98.00

*TP* true positive, *FP* false positive, *TN* true negative, *FN* false negative

Accuracy = (TP + TN)/(TP + FN + TN + FP); Sensitivity = TP/(TP + FN); Specificity = TN/(TN + FP)

Average accuracy: 95.29%

The average prediction accuracy of the classifier was 95.29% in a completely independent set of 108 samples. In addition, we observed that the prediction accuracy for the “Others” and “Hyperdiploid” subtypes was lower compared with other subtypes. These data are consistent with the fact that these two subtypes are heterogeneous subgroups and should have identifiable genetic abnormalities. The classifier achieved a 98.28% average accuracy without those samples. These findings indicated that the classifier performed well for the iAFA data.

### Marker gene selection for prognostic value

**Fig. 4:**
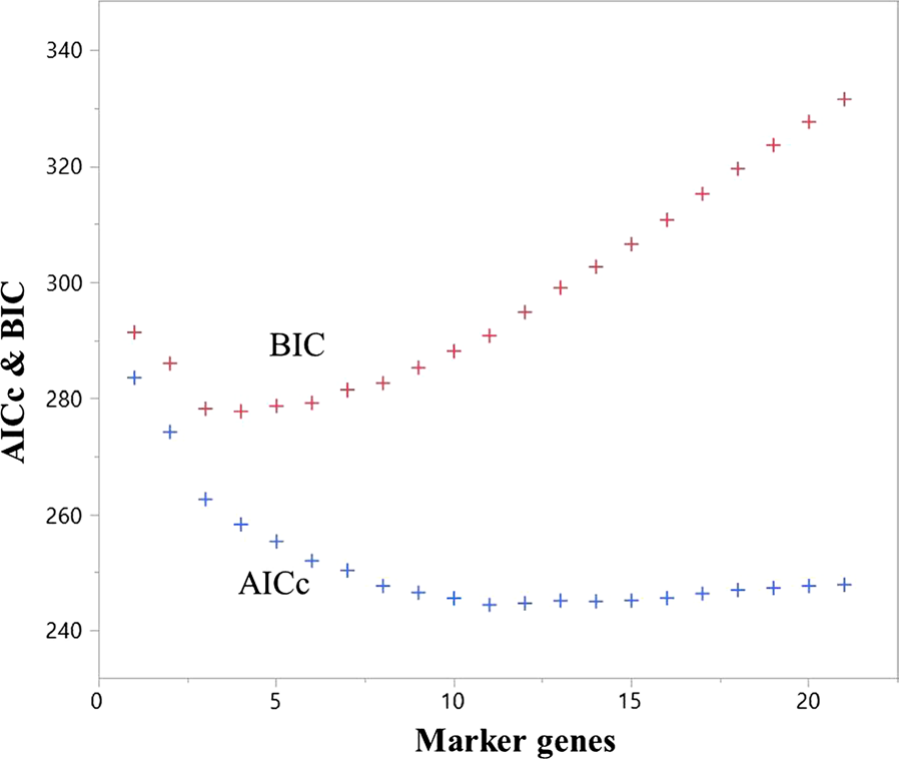
Relationship between the number of marker genes and the AICc & BIC. The corrected Akaike’s information criterion (AICc) and the Bayesian information criterion (BIC) are information-based criteria that assess model fit

We also investigated the prognostic significance of the marker genes. An increase in the number of marker genes causes the AICc to decrease gradually. However, with 10 marker genes, the AICc hardly decreases and slightly increases with 13 marker genes. On the other hand, the BIC decreases from the start and increases when the number of marker genes is 4 (Fig. 4). This indicates that the goodness of fit of the model may become worse as the number of marker genes increases.

According to the AICc and BIC, we chose 10 candidate models from 4 to 13 marker genes. Logistic analysis with fivefold cross validation showed that the model with 10 marker genes had the lowest misclassification rate in the validation set (Table 2) and was therefore considered the optimal prediction model. The parameter estimation of the model is shown in Additional file 1: Table S4.

**Table 2:** Error rate of each candidate prediction model

No.	Marker genes	Error rate
4	*ITM2A COL6A3 PTPRK RBMS1*	11.64
5	*ITM2A COL6A3 PTPRK RBMS1 IMP*-*3*	10.58
6	*ITM2A COL6A3 PTPRK RBMS1 IMP*-*3 ALOX5*	11.11
7	*ITM2A COL6A3 PTPRK RBMS1 IMP*-*3 ALOX5 CD69*	10.85
8	*ITM2A COL6A3 PTPRK RBMS1 IMP*-*3 ALOX5 CD69 STCH*	10.85
9	*ITM2A COL6A3 PTPRK RBMS1 IMP*-*3 ALOX5 CD69 STCH FNDC3A*	10.85
10	*ITM2A COL6A3 PTPRK RBMS1 IMP*-*3 ALOX5 CD69 STCH FNDC3A PROM1*	10.32
11	*ITM2A COL6A3 PTPRK RBMS1 IMP*-*3 ALOX5 CD69 STCH FNDC3A PROM1 MARCKS*	10.58
12	*ITM2A COL6A3 PTPRK RBMS1 IMP*-*3 ALOX5 CD69 STCH FNDC3A PROM1 MARCKS ITGA6*	10.58
13	*ITM2A COL6A3 PTPRK RBMS1 IMP*-*3 ALOX5 CD69 STCH FNDC3A PROM1 MARCKS ITGA6 FRMD4B*	10.85

### The prognostic significance of 10 marker genes in pediatric ALL

**Fig. 5:**
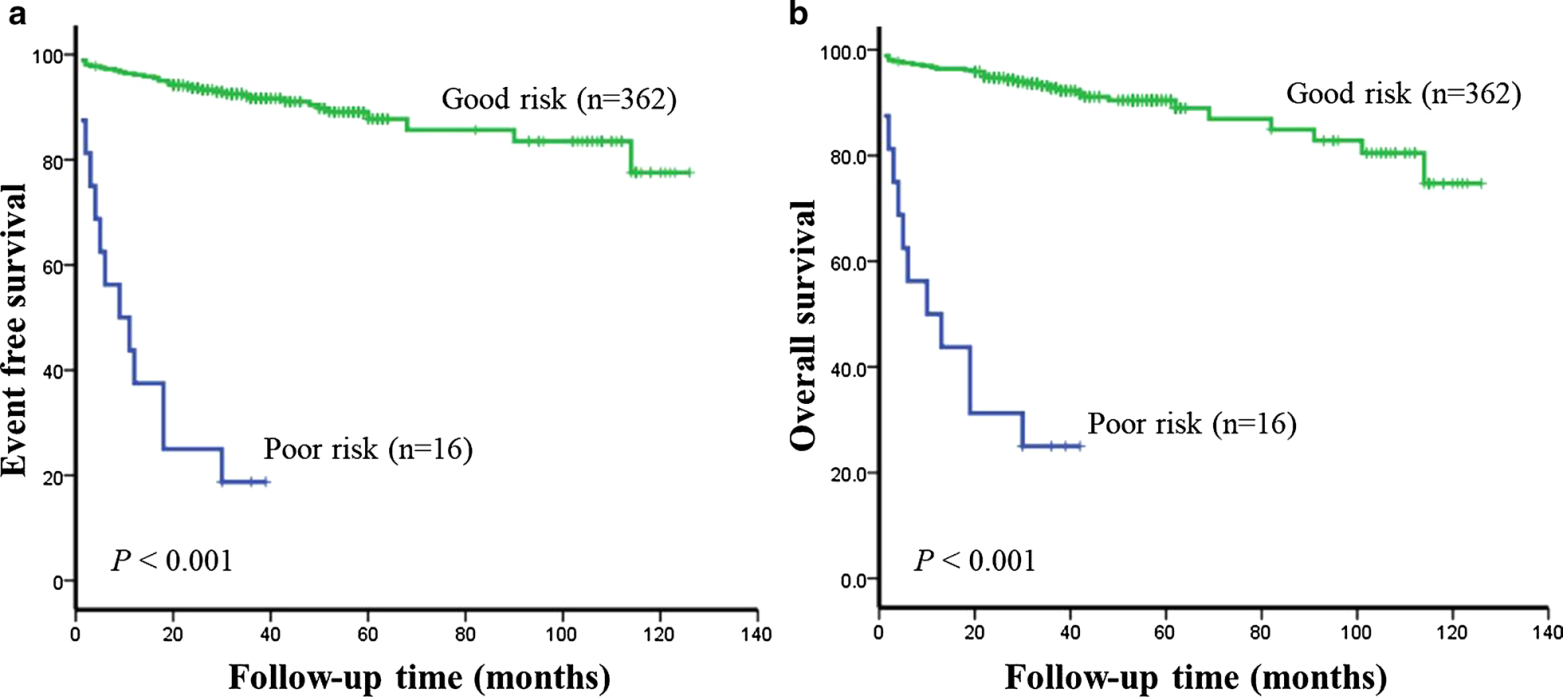
The prognostic significance of 10 marker genes in 378 pediatric ALL patients. **a** Event-free survival. **b** Overall survival

Because of the small number of iAFA samples and the short follow-up time, the previous 160 AFA data were analyzed along with 219 iAFA data, and 27 patients had a dismal prognosis (Additional file 1: Table S5). Except for one child whose prognostic information was unable to be tracked, 378 patients were divided into 2 genetic risk groups: the good-risk (GR) group and the poor-risk (PR) group. Respectively, these 2 genetic risk groups consisted of 95.8% and 4.2% of the cases and were associated with significantly different EFS rates at 5 years, 93.6 ± 1.3% vs 18.8 ± 9.8% (Fig. 5a), and different OS rates at 5 years, 94.7 ± 1.2% vs 25.0 ± 10.8% (Fig. 5b).

**Fig. 6:**
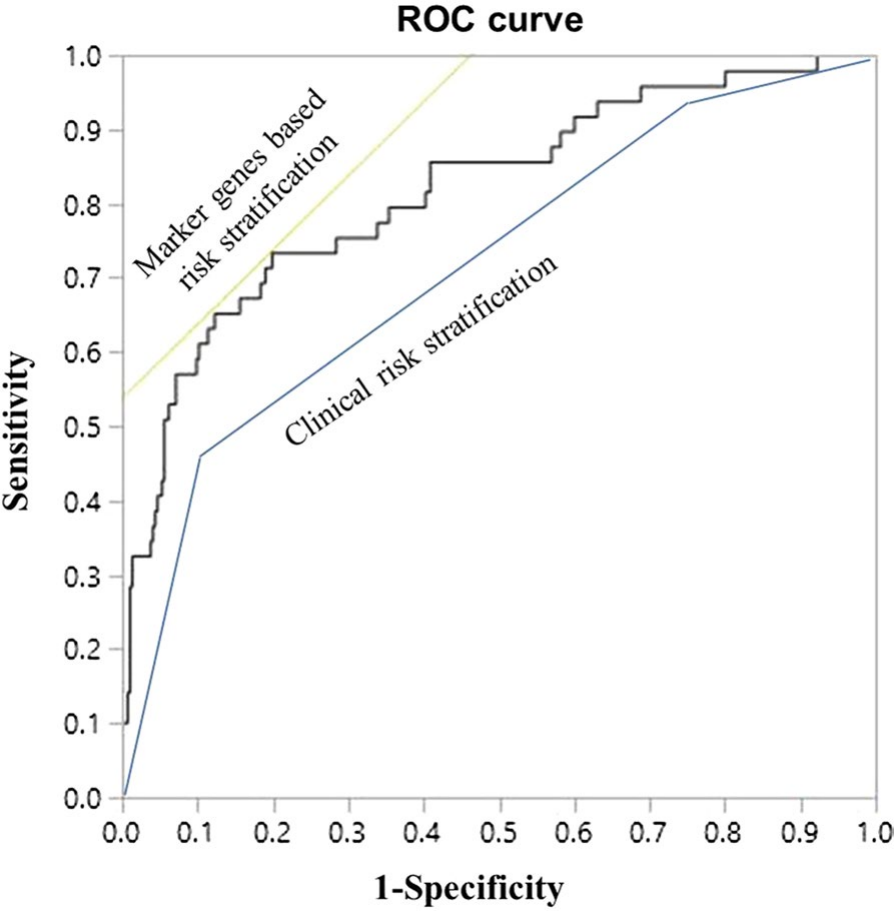
Comparison of the prognostic predictive value of risk stratification in 378 children with ALL. The AUC for clinical risk stratification and gene expression-based risk stratification were 0.7040 and 0.8191, respectively

The predictive value of 10 marker genes was tested using a receiver operating characteristic (ROC) curve. The area under the curve (AUC) was 0.8191 (*P* < 0.001), which was better than that of our current clinical risk stratification (0.7040, Fig. 6). We also investigated the association of the 10 marker gene expression profiles with common clinical characteristics. There was no difference only in gender within the marker gene expression-based risk group (*P *> 0.05). As we expected, the expression of these 10 marker genes was significantly associated with adverse clinical risk factors, such as age, white blood cell count in peripheral blood at diagnosis, immunophenotype, chromosome abnormalities in B-ALL, prednisone response, our current clinical risk stratification, treatment outcome and MRD at day 33 and day 78 (Table 3). These results indicated that these 10 marker genes have a strong prognostic significance.

**Table 3:** Comparison of common clinical characteristics according to the 10 marker gene expression-based risk groups

Variable/category	Gene expression-based risk group		χ^2^	*P* value
	GR group n (%)	PR group n (%)		
Total number of patients	362 (95.8)	16 (4.2)		
Age (year)				
≥ 10	41 (11.3)	2 (12.5)	6.75	0.031
1–10	316 (87.3)	12 (75.0)		
< 1	5 (1.4)	2 (12.5)		
Gender				
Male	229 (63.3)	9 (56.2)	0.32	0.570
Female	133 (36.7)	7 (43.8)		
WBC (× 10^9^/l)				
≥ 50 × 10^9^/l	72 (19.9)	12 (75.0)	23.83	< 0.001
< 50 × 10^9^/l	290 (80.1)	4 (25.0)		
Immunophenotype				
T-ALL	29 (8.0)	7 (43.8)	18.76	< 0.001
B-ALL	333 (92.0)	9 (56.2)		
Chromosome abnormalities in B-ALL				
*BCR*-*ABL1*	20 (6.0)	6 (66.7)	31.22	< 0.001
*E2A*-*PBX1*	27 (8.1)	0		
*TEL*-*AML1*	109 (32.7)	0		
*MLL* rearrangements	8 (2.4)	2 (22.2)		
Hyperdiploid > 50	107 (32.1)	0		
Other B-ALL	62 (18.6)	1 (11.1)		
Prednisone response				
Good	351 (97.0)	11 (68.8)	23.53	< 0.001
Poor	11 (3.0)	5 (31.2)		
MRD at day 33				
Positive	12 (3.6)	3 (27.3)	9.04	0.003
Negative	317 (96.4)	8 (72.7)		
Not evaluated	33	5		
MRD at day 78				
Positive	7 (2.2)	2 (22.2)	6.69	0.010
Negative	311 (97.8)	7 (77.8)		
Not evaluated	44	7		
Clinical risk group				
Standard risk	87 (24.0)	0	30.83	< 0.001
Intermediate risk	230 (63.5)	4 (25.0)		
High risk	45 (12.4)	12 (75.0)		
Outcome				
Event^a^	36 (9.9)	13 (81.3)	62.88	< 0.001
Remission	326 (90.1)	3 (18.7)		

^a^Event: ALL relapse, second malignant neoplasms (SMNs) or death

## Discussion

Pediatric ALL comprises multiple entities with distinct genetic alterations, clinical characteristics, treatment protocols and prognosis features [17–19]. Accurate diagnostic classification and risk stratification are critical for the administration of the appropriate therapy and avoiding unnecessary treatment-related complications in patients.

One-step multiplex RT-PCR assay and UDG enzyme-based anti-contamination system were used to improve amplification assays, which have been widely applied to the identification of a variety of viruses in recent years [20–25]. We opted to combine the above two pragmatic approaches with the original AFA technique to deliver a simpler and more rapid iAFA technology that could be readily adopted into routine clinical practice for ALL classification.

The iAFA technique saves 1 h over the whole operation process compared with the previous AFA assay. A one-step multiplex RT-PCR assay is easier to operate with high sensitivity [20]. Contamination usually occurs by opening the reaction tubes, leading to aerosol droplets of different sizes that contain high concentrations of amplicons [26]. The UDG enzyme digests carryover contamination via the following two steps: (i) incorporating dUTP in all amplification products (by substituting dUTP for dTTP); and (ii) treating all subsequent, fully preassembled starting reactions with the UDG enzyme, followed by thermal inactivation of the UDG enzyme. The enzyme cleaves the uracil base from the phosphodiester backbone of uracil-containing DNA but has no effect on natural (i.e., thymine-containing) DNA or RNA templates [27]. In this assay, the UDG enzyme process was performed before reaction at 25 °C to eliminate carryover contamination and then deactivated at 50 °C due to its heat-sensitive characteristics.

In this study, a new classifier based on iAFA data has been developed that can classify pediatric ALL patients into the most common 7 subtypes. The prediction accuracy for the “Others” and “Hyperdiploid” subtypes was lower compared with other subtypes. This phenomenon coincides with results from previous ALL studies in which these two subtypes are heterogeneous subgroups and should have subtle identifiable genetic abnormalities [8, 28]. There are strengths and limitations to our study compared with other attempts to develop or refine risk classification. First, this classifier is similar to one we previously constructed, and it can also take a single sample and make a prediction based solely on the relative expression ranks among the marker genes without consulting the signal distribution of other parallel-processed samples. This characteristic is not only suitable for dealing with brand new clinical ALL samples but also applies to analyzing a large number of samples for scientific research. Second, our classifier is thus far a relatively rapid, classifiable method using the gene expression profile of pediatric ALL, which fulfills precisely the urgent need for clinical classification and reasonable treatment. However, one potential limitation to our work is that only the most common 7 subtypes with current clinical relevance are covered, and not all novel recurrent cytogenetic abnormalities of pediatric ALL were observed in our cases, especially some relatively rare but significant genetic alterations such as *IKZF1* deletions, intrachromosomal amplification of chromosome 21 (iAMP21), low hypodiploidy (30–39 chromosomes), *JAK2* mutations, fusion genes involving *ZNF384*, *CRLF2* rearrangements, and the *MEF2D*-*BCL9* fusion gene [18, 29–33]. Further work is required to enlarge the sample size and build a comprehensive classifier.

The AUC of the ROC curve of our current clinical risk stratification was only 0.7040 in this study, perhaps not ideal because individual treatment was carried out according to CCLG-2008 risk stratification. High-risk children may not appear with a dismal prognosis, including ALL relapse, SMNs and death. However, the diagnostic efficacy of clinical risk stratification remains to be further improved. As expected, the 10 marker gene expression profile was able to predict treatment outcome more precisely than current clinical risk stratification. This 10 marker gene expression-based risk stratification defines a large (95.8%) subset of patients who have an excellent outcome (5-year EFS rates exceeding 90%). Only 4.2% of patients were classified as PR based on the marker gene expression profile. The EFS of PR patients was less than 20%, significantly inferior to GR patients. Further improvements in the treatment of pediatric ALL cannot be measured only in terms of relapse rate and death toll [29]. Instead, reducing the frequency and severity of long-term toxicities without adversely affecting cure rates is becoming the major focus of treatment [34]. Hence, future treatment strategies for most patients with an excellent outcome should focus on deintensification to avoid side effects. For extremely high-risk children, more intensified treatment, hematopoietic stem-cell transplantation, and molecular-targeted therapy may improve their prognosis [35]. Further work is required to follow-up on all the patients, validate the new risk stratification on a completely independent cohort of patients and optimize the therapeutic regimen.

## Conclusion

In conclusion, we integrated a one-step multiplex RT-PCR assay and an UDG enzyme-based anti-contamination system into the original AFA technique. It serves as a convenient and reliable classification and risk stratification. It may add a new layer to current diagnostic classification and could offer a reliable platform for patients who lack access to current state-of-the-art diagnostic work-up. Future refinements need to include additional signatures for prognostically important subsets of patients with ALL.

## Additional file

**Additional file 1: Table S1.** Clinical features of pediatric ALL cases for bone marrow samples. **Table S2.** Primers of the marker genes in the improved AFA multiplex assay. **Table S3.** Prediction results for 219 iAFA samples. **Table S4.** Parameter estimation of the optimal prediction model. **Table S5.** Twenty-seven children in the previous 160 ALL cases with dismal prognosis.

## Abbreviations

AFA: advanced fragment analysis
AICc: corrected Akaike’s information criterion
ALL: acute lymphoblastic leukemia
AUC: area under the curve
BIC: Bayesian information criterion
BM: bone marrow
CCLG: China Children’s Leukemia Group
EFS: event-free survival
GR: good-risk
iAFA: improved advanced fragment analysis
ID: initial diagnosis
MRD: minimal residual disease
OS: overall survival
PR: poor-risk
SMNs: second malignant neoplasms
UDG: uracil-DNA glycosylase
