# Supplementary material for: An improved advanced fragment analysis-based classification and risk stratification of pediatric acute lymphoblastic leukemia
Source: Cancer Cell Int. 2019 Apr 25;19:110. doi: 10.1186/s12935-019-0825-y (PMC6482565; doi:10.1186/s12935-019-0825-y)
Supplement: Supplementary file 1 — Additional file 1: Table S1. Clinical features of pediatric ALL cases for bone marrow samples. Table S2. Primers of the marker genes in the improved AFA multiplex assay. Table S3. Prediction results for 219 iAFA samples. Table S4. Parameter estimation of the optimal prediction model. Table S5. Twenty-seven children in the previous 160 ALL cases with dismal prognosis. [file 12935_2019_825_MOESM1_ESM.docx]

**Table S1**

**Clinical features of pediatric ALL cases for bone marrow samples**

| **NO.** | **Sex** | **Age of diagnosis (years)** | **WBC in PB at ID (×10^9^/L)** | **Percentage of blast cells in BM at ID (%)** | **Immunotype** | **Cytogenetic abnormality** | **Fusion gene** | **Outcome** |
| --- | --- | --- | --- | --- | --- | --- | --- | --- |
| 1 | F | 2 | 8.54 | 89.5 | c-B-ALL | t(1;19)(q23;p13) | *E2A-PBX1* | Remission |
| 2 | M | 10 | 20.23 | 90 | c-B-ALL | t(1;19)(q23;p13) | *E2A-PBX1* | Remission |
| 3 | F | 10 | 15.19 | 84.5 | c-B-ALL | t(1;19)(q23;p13) | *E2A-PBX1* | Remission |
| 4 | M | 1 | 22.36 | 96 | c-B-ALL | t(1;19)(q23;p13) | *E2A-PBX1* | Remission |
| 5 | M | 12 | 98.97 | 80.5 | c-B-ALL | t(1;19)(q23;p13) | *E2A-PBX1* | Remission |
| 6 | F | 4 | 82.34 | 94.5 | c-B-ALL | t(1;19)(q23;p13) | *E2A-PBX1* | Remission |
| 7 | M | 10 | 14.54 | 85.5 | c-B-ALL | t(1;19)(q23;p13) | *E2A-PBX1* | Remission |
| 8 | M | 4 | 23.9 | 97 | c-B-ALL | t(1;19)(q23;p13) | *E2A-PBX1* | Remission |
| 9 | F | 8 | 21.6 | 96 | c-B-ALL | t(1;19)(q23;p13) | *E2A-PBX1* | Death |
| 10 | F | 4 | 12.31 | 77 | c-B-ALL | t(1;19)(q23;p13) | *E2A-PBX1* | Death |
| 11 | F | 2 | 11.32 | 76 | c-B-ALL | t(1;19)(q23;p13) | *E2A-PBX1* | Remission |
| 12 | M | 9 | 8 | 71 | c-B-ALL | t(1;19)(q23;p13) | *E2A-PBX1* | Remission |
| 13 | F | 11 | 11.9 | 98 | c-B-ALL | t(12;21)(p13;q22) | *TEL-AML1* | Remission |
| 14 | M | 9 | 2.48 | 93.5 | c-B-ALL | t(12;21)(p13;q22) | *TEL-AML1* | Death |
| 15 | F | 2 | 2.71 | 86 | c-B-ALL | t(12;21)(p13;q22) | *TEL-AML1* | Remission |
| 16 | F | 13 | 53.1 | 91 | c-B-ALL | t(12;21)(p13;q22) | *TEL-AML1* | Remission |
| 17 | M | 5 | 60.26 | 96 | c-B-ALL | t(12;21)(p13;q22) | *TEL-AML1* | Remission |
| 18 | F | 8 | 9.79 | 95 | c-B-ALL | t(12;21)(p13;q22) | *TEL-AML1* | Remission |
| 19 | F | 3 | 5.54 | 50 | c-B-ALL | t(12;21)(p13;q22) | *TEL-AML1* | Remission |
| 20 | F | 3 | 51.01 | 98 | c-B-ALL | t(12;21)(p13;q22) | *TEL-AML1* | Remission |
| 21 | F | 3 | 6.72 | 90 | c-B-ALL | t(12;21)(p13;q22) | *TEL-AML1* | Remission |
| 22 | M | 6 | 9.93 | 95 | c-B-ALL | t(12;21)(p13;q22) | *TEL-AML1* | Remission |
| 23 | F | 7 | 5.59 | 92.5 | c-B-ALL | t(12;21)(p13;q22) | *TEL-AML1* | Remission |
| 24 | F | 3 | 12.98 | 90.5 | c-B-ALL | t(12;21)(p13;q22) | *TEL-AML1* | Remission |
| 25 | M | 3 | 15.08 | 94 | c-B-ALL | t(12;21)(p13;q22) | *TEL-AML1* | Remission |
| 26 | F | 4 | 3.94 | 90.5 | c-B-ALL | t(12;21)(p13;q22) | *TEL-AML1* | Relapse |
| 27 | M | 3 | 95.03 | 93.5 | c-B-ALL | t(12;21)(p13;q22) | *TEL-AML1* | Remission |
| 28 | M | 2 | 7.48 | 96 | c-B-ALL | t(12;21)(p13;q22) | *TEL-AML1* | Remission |
| 29 | M | 2 | 3.88 | 97 | c-B-ALL | t(12;21)(p13;q22) | *TEL-AML1* | Remission |
| 30 | F | 7 | 2.84 | 94 | c-B-ALL | t(12;21)(p13;q22) | *TEL-AML1* | Remission |
| 31 | M | 2 | 3.74 | 94.5 | c-B-ALL | t(12;21)(p13;q22) | *TEL-AML1* | Death |
| 32 | M | 2 | 13.51 | 93.5 | c-B-ALL | t(12;21)(p13;q22) | *TEL-AML1* | Remission |
| 33 | M | 2 | 69.1 | 94.5 | c-B-ALL | t(12;21)(p13;q22) | *TEL-AML1* | Remission |
| 34 | M | 3 | 7.04 | 63 | c-B-ALL | t(12;21)(p13;q22) | *TEL-AML1* | Remission |
| 35 | M | 3 | 4.61 | 96.5 | c-B-ALL | t(12;21)(p13;q22) | *TEL-AML1* | Remission |
| 36 | F | 3 | 9.83 | 97 | c-B-ALL | t(12;21)(p13;q22) | *TEL-AML1* | Remission |
| 37 | F | 3 | 99.7 | 97 | c-B-ALL | t(12;21)(p13;q22) | *TEL-AML1* | Remission |
| 38 | F | 6 | 1.58 | 88.5 | c-B-ALL | t(12;21)(p13;q22) | *TEL-AML1* | Remission |
| 39 | F | 3 | 9.07 | 98 | c-B-ALL | t(12;21)(p13;q22) | *TEL-AML1* | Remission |
| 40 | F | 2 | 6.12 | 91 | c-B-ALL | t(12;21)(p13;q22) | *TEL-AML1* | Remission |
| 41 | M | 3 | 6.39 | 98 | c-B-ALL | t(12;21)(p13;q22) | *TEL-AML1* | Remission |
| 42 | F | 10 | 2.6 | 92 | c-B-ALL | t(12;21)(p13;q22) | *TEL-AML1* | Remission |
| 43 | F | 2 | 205.1 | 93 | c-B-ALL | t(12;21)(p13;q22) | *TEL-AML1* | Remission |
| 44 | F | 5 | 40.6 | 96.5 | c-B-ALL | t(12;21)(p13;q22) | *TEL-AML1* | Remission |
| 45 | M | 3 | 7.25 | 84.5 | c-B-ALL | t(12;21)(p13;q22) | *TEL-AML1* | Remission |
| 46 | M | 3 | 6.32 | 91.5 | c-B-ALL | t(12;21)(p13;q22) | *TEL-AML1* | Remission |
| 47 | F | 8 | 12.97 | 53 | c-B-ALL | t(12;21)(p13;q22) | *TEL-AML1* | Remission |
| 48 | M | 9 | 3.48 | 50.5 | c-B-ALL | t(12;21)(p13;q22) | *TEL-AML1* | Remission |
| 49 | M | 4 | 3.1 | 96.5 | c-B-ALL | t(12;21)(p13;q22) | *TEL-AML1* | Remission |
| 50 | M | 8 | 78.53 | 89.5 | c-B-ALL | t(12;21)(p13;q22) | *TEL-AML1* | Remission |
| 51 | F | 2 | 7.6 | 64 | c-B-ALL | t(12;21)(p13;q22) | *TEL-AML1* | Remission |
| 52 | M | 2 | 165.47 | 97.5 | c-B-ALL | t(12;21)(p13;q22) | *TEL-AML1* | Remission |
| 53 | M | 6 | 4.06 | 93 | c-B-ALL | t(12;21)(p13;q22) | *TEL-AML1* | Remission |
| 54 | M | 4 | 14.19 | 88.5 | c-B-ALL | t(12;21)(p13;q22) | *TEL-AML1* | Remission |
| 55 | M | 3 | 18.15 | 95.5 | c-B-ALL | t(12;21)(p13;q22) | *TEL-AML1* | Remission |
| 56 | F | 4 | 22.22 | 90 | c-B-ALL | t(12;21)(p13;q22) | *TEL-AML1* | Remission |
| 57 | F | 2 | 7.7 | 79.5 | c-B-ALL | t(12;21)(p13;q22) | *TEL-AML1* | Remission |
| 58 | M | 2 | 7.26 | 87.5 | c-B-ALL | t(12;21)(p13;q22) | *TEL-AML1* | Remission |
| 59 | F | 3 | 9.88 | 96.5 | c-B-ALL | t(12;21)(p13;q22) | *TEL-AML1* | Remission |
| 60 | M | 5 | 18.79 | 44.8 | c-B-ALL | t(12;21)(p13;q22) | *TEL-AML1* | Remission |
| 61 | F | 6 | 9.07 | 77.5 | c-B-ALL | t(12;21)(p13;q22) | *TEL-AML1* | Remission |
| 62 | F | 7 | 117.18 | 91 | c-B-ALL | t(12;21)(p13;q22) | *TEL-AML1* | Remission |
| 63 | M | 6 | 71.37 | 94 | c-B-ALL | t(12;21)(p13;q22) | *TEL-AML1* | Remission |
| 64 | F | 2 | 139.28 | 97 | c-B-ALL | t(12;21)(p13;q22) | *TEL-AML1* | Remission |
| 65 | M | 3 | 4.59 | 90.5 | c-B-ALL | t(12;21)(p13;q22) | *TEL-AML1* | Remission |
| 66 | M | 3 | 10.1 | 96 | c-B-ALL | t(12;21)(p13;q22) | *TEL-AML1* | Remission |
| 67 | M | 3 | 4.38 | 93.5 | c-B-ALL | t(12;21)(p13;q22) | *TEL-AML1* | Remission |
| 68 | F | 5 | 3.38 | 94 | c-B-ALL | t(12;21)(p13;q22) | *TEL-AML1* | Remission |
| 69 | F | 0.83 | 15.26 | 88 | c-B-ALL | t(9;11)(p22;q23) | *MLL-AF9* | Death |
| 70 | F | 0.42 | 88.7 | 96 | c-B-ALL | t(11;19)(q23;p13) | *MLL-ENL* | Relapse |
| 71 | F | 0.58 | 96.26 | 91 | c-B-ALL | t(9;11)(p22;q23) | *MLL-AF9* | Relapse |
| 72 | M | 2 | 45.34 | 96 | pro-B-ALL | t(4;11)(q21;q23) | *MLL-AF4* | Remission |
| 73 | F | 0.83 | 25.18 | 91 | pro-B-ALL | t(4;11)(q21;q23) | *MLL-AF4* | Death |
| 74 | M | 9 | 6.38 | 89.5 | c-B-ALL | t(9;22)(q34;q11) | *BCR-ABL1* | Remission |
| 75 | F | 4 | 133.16 | 87 | c-B-ALL | t(9;22)(q34;q11) | *BCR-ABL1* | Remission |
| 76 | M | 13 | 73.72 | 95 | c-B-ALL | t(9;22)(q34;q11) | *BCR-ABL1* | Remission |
| 77 | F | 9 | 13.98 | 83.5 | c-B-ALL | t(9;22)(q34;q11) | *BCR-ABL1* | Remission |
| 78 | M | 3 | 94.65 | 94 | c-B-ALL | t(9;22)(q34;q11) | *BCR-ABL1* | Remission |
| 79 | M | 4 | 57.2 | 79 | c-B-ALL | t(9;22)(q34;q11) | *BCR-ABL1* | Remission |
| 80 | M | 3 | 715.25 | 94.5 | c-B-ALL | t(9;22)(q34;q11) | *BCR-ABL1* | Remission |
| 81 | F | 6 | 215.25 | 97 | c-B-ALL | t(9;22)(q34;q11) | *BCR-ABL1* | Remission |
| 82 | M | 4 | 5.09 | 96.5 | c-B-ALL | t(9;22)(q34;q11) | *BCR-ABL1* | Remission |
| 83 | M | 8 | 1.28 | 97.5 | c-B-ALL | Hyperdiploid | / | Remission |
| 84 | F | 4 | 60.85 | 96 | c-B-ALL | Hyperdiploid | / | Remission |
| 85 | F | 6 | 1.28 | 78 | c-B-ALL | Hyperdiploid | / | Remission |
| 86 | M | 2 | 2.05 | 96.5 | c-B-ALL | Hyperdiploid | / | Remission |
| 87 | F | 4 | 3.97 | 92 | c-B-ALL | Hyperdiploid | / | Remission |
| 88 | M | 2 | 43.93 | 94 | c-B-ALL | Hyperdiploid | / | Death |
| 89 | F | 2 | 31.94 | 95 | c-B-ALL | Hyperdiploid | / | Remission |
| 90 | F | 4 | 7.07 | 96 | c-B-ALL | Hyperdiploid | / | Remission |
| 91 | M | 5 | 3.21 | 87 | c-B-ALL | Hyperdiploid | / | Remission |
| 92 | F | 5 | 7.52 | 74.5 | c-B-ALL | Hyperdiploid | / | Death |
| 93 | M | 3 | 14.86 | 98 | c-B-ALL | Hyperdiploid | / | Remission |
| 94 | M | 3 | 19.2 | 95 | c-B-ALL | Hyperdiploid | / | Remission |
| 95 | M | 6 | 26.98 | 98 | c-B-ALL | Hyperdiploid | / | Relapse |
| 96 | M | 6 | 3.71 | 89 | c-B-ALL | Hyperdiploid | / | Remission |
| 97 | M | 5 | 13.47 | 70.5 | pre-B-ALL | Hyperdiploid | / | Remission |
| 98 | F | 6 | 11.45 | 97.5 | c-B-ALL | Hyperdiploid | / | Remission |
| 99 | F | 12 | 5.87 | 91.5 | c-B-ALL | Hyperdiploid | / | Remission |
| 100 | F | 6 | 4.98 | 91 | c-B-ALL | Hyperdiploid | / | Remission |
| 101 | F | 5 | 15.12 | 97 | pre-B-ALL | Hyperdiploid | / | Remission |
| 102 | F | 11 | 4.28 | 95 | c-B-ALL | Hyperdiploid | / | Remission |
| 103 | F | 3 | 2.56 | 85 | c-B-ALL | Hyperdiploid | / | Remission |
| 104 | M | 4 | 8.06 | 94 | c-B-ALL | Hyperdiploid | / | Remission |
| 105 | F | 2 | 27.78 | 98 | c-B-ALL | Hyperdiploid | / | Relapse |
| 106 | M | 3 | 1.04 | 94 | c-B-ALL | Hyperdiploid | / | Remission |
| 107 | M | 4 | 10.88 | 94.5 | c-B-ALL | Hyperdiploid | / | Remission |
| 108 | M | 1 | 5.56 | 92.5 | c-B-ALL | Hyperdiploid | / | Remission |
| 109 | M | 5 | 7.25 | 71 | c-B-ALL | Hyperdiploid | / | Remission |
| 110 | M | 2 | 56.51 | 94.5 | c-B-ALL | Hyperdiploid | / | Remission |
| 111 | M | 2 | 1.73 | 85 | c-B-ALL | Hyperdiploid | / | Remission |
| 112 | M | 5 | 13.59 | 88.5 | c-B-ALL | Hyperdiploid | / | Remission |
| 113 | M | 4 | 2.68 | 83.5 | c-B-ALL | Hyperdiploid | / | Remission |
| 114 | M | 3 | 57.32 | 94 | c-B-ALL | Hyperdiploid | / | Death |
| 115 | F | 3 | 5.49 | 94.5 | c-B-ALL | Hyperdiploid | / | Remission |
| 116 | F | 6 | 59 | 98.5 | c-B-ALL | Hyperdiploid | / | Remission |
| 117 | M | 8 | 3.46 | 93 | c-B-ALL | Hyperdiploid | / | Remission |
| 118 | M | 3 | 14.53 | 97 | c-B-ALL | Hyperdiploid | / | Remission |
| 119 | M | 2 | 50.64 | 97 | c-B-ALL | Hyperdiploid | / | Remission |
| 120 | F | 2 | 4.09 | 94 | c-B-ALL | Hyperdiploid | / | Remission |
| 121 | M | 3 | 3.09 | 97.5 | c-B-ALL | Hyperdiploid | / | Remission |
| 122 | M | 2 | 11.97 | 85 | c-B-ALL | Hyperdiploid | / | Remission |
| 123 | M | 2 | 5.17 | 98 | c-B-ALL | Hyperdiploid | / | Remission |
| 124 | M | 5 | 11.74 | 98 | c-B-ALL | Hyperdiploid | / | Remission |
| 125 | F | 2 | 87.02 | 95 | c-B-ALL | Hyperdiploid | / | Remission |
| 126 | F | 1 | 8.8 | 96.5 | c-B-ALL | Hyperdiploid | / | Remission |
| 127 | M | 2 | 4.57 | 96 | c-B-ALL | Hyperdiploid | / | Remission |
| 128 | M | 2 | 3.29 | 97 | c-B-ALL | Hyperdiploid | / | Remission |
| 129 | F | 3 | 5.15 | 95 | c-B-ALL | Hyperdiploid | / | Remission |
| 130 | M | 2 | 4.09 | 91.5 | c-B-ALL | Hyperdiploid | / | Remission |
| 131 | M | 3 | 3.87 | 79 | c-B-ALL | Hyperdiploid | / | Remission |
| 132 | M | 4 | 4.08 | 98 | c-B-ALL | Hyperdiploid | / | Remission |
| 133 | F | 3 | 22.47 | 98.5 | c-B-ALL | Hyperdiploid | / | Remission |
| 134 | F | 9 | 11.33 | 92 | c-B-ALL | Hyperdiploid | / | Remission |
| 135 | M | 1 | 54.02 | 98 | c-B-ALL | Hyperdiploid | / | Remission |
| 136 | M | 3 | 3.03 | 87 | c-B-ALL | Hyperdiploid | / | Remission |
| 137 | M | 3 | 9.11 | 93.5 | c-B-ALL | Hyperdiploid | / | Remission |
| 138 | M | 5 | 3.18 | 70 | c-B-ALL | Hyperdiploid | / | Remission |
| 139 | F | 2 | 7.94 | 96.5 | c-B-ALL | Hyperdiploid | / | Remission |
| 140 | M | 4 | 3.76 | 92.5 | c-B-ALL | Hyperdiploid | / | Remission |
| 141 | F | 2 | 4.58 | 90 | c-B-ALL | Hyperdiploid | / | Remission |
| 142 | M | 4 | 6.35 | 92 | c-B-ALL | Hyperdiploid | / | Remission |
| 143 | M | 4 | 3.19 | 91.5 | c-B-ALL | Hyperdiploid | / | Remission |
| 144 | F | 3 | 10.31 | 93.5 | c-B-ALL | Hyperdiploid | / | Remission |
| 145 | M | 2 | 8.3 | 86.5 | c-B-ALL | Hyperdiploid | / | Remission |
| 146 | M | 1 | 2.98 | 98.5 | c-B-ALL | Hyperdiploid | / | Remission |
| 147 | F | 4 | 52 | 87.5 | c-B-ALL | Hyperdiploid |  | Remission |
| 148 | F | 3 | 14.58 | 96 | c-B-ALL | Hyperdiploid | / | Remission |
| 149 | M | 2 | 83.3 | 91.5 | c-B-ALL | Hyperdiploid | / | Remission |
| 150 | F | 3 | 55.27 | 92 | c-B-ALL | Hyperdiploid | / | Remission |
| 151 | F | 1 | 1.06 | 89.5 | c-B-ALL | Hyperdiploid | / | Remission |
| 152 | F | 4 | 8.8 | 90 | c-B-ALL | Hyperdiploid | / | Remission |
| 153 | M | 3 | 6.07 | 89.5 | c-B-ALL | Hyperdiploid | / | Remission |
| 154 | F | 7 | 2.68 | 88.5 | T-ALL | / | / | Remission |
| 155 | F | 10 | 55.43 | 90.5 | T-ALL | / | / | Remission |
| 156 | M | 2 | 66.17 | 76 | T-ALL | / | / | Remission |
| 157 | M | 11 | 111.3 | 82.5 | T-ALL | / | / | Remission |
| 158 | M | 7 | 37.88 | 87 | T-ALL | / | / | Remission |
| 159 | M | 4 | 115.84 | 63.5 | T-ALL | / | / | Remission |
| 160 | M | 13 | 490 | 95.5 | T-ALL | / | / | Remission |
| 161 | F | 5 | 341.69 | 94 | T-ALL | / | / | Relapse |
| 162 | F | 1 | 92.82 | 92.5 | T-ALL | / | / | Remission |
| 163 | M | 9 | 122.05 | 86 | T-ALL | / | / | Remission |
| 164 | M | 14 | 68.23 | 53 | T-ALL | / | / | Remission |
| 165 | F | 8 | 391 | 91 | T-ALL | del(1)(p34p34) | *SIL-TAL1* | Relapse |
| 166 | M | 1 | 223 | 79 | T-ALL | del(1)(p34p34) | *SIL-TAL1* | Remission |
| 167 | M | 1 | 123.83 | 92 | T-ALL | del(1)(p34p34) | *SIL-TAL1* | Remission |
| 168 | M | 1 | 95.18 | 92.5 | T-ALL | del(1)(p34p34) | *SIL-TAL1* | Death |
| 169 | M | 8 | 91.71 | 88.5 | T-ALL | / | / | Remission |
| 170 | M | 1 | 52.29 | 85.5 | pro-B-ALL | / | / | Remission |
| 171 | M | 2 | 9.33 | 95 | c-B-ALL | / | / | Remission |
| 172 | M | 13 | 5.85 | 85 | c-B-ALL | / | / | Remission |
| 173 | F | 10 | 1.62 | 95 | c-B-ALL | / | / | Remission |
| 174 | M | 6 | 5.18 | 91 | c-B-ALL | / | / | Remission |
| 175 | M | 6 | 4.9 | 96 | c-B-ALL | / | / | Remission |
| 176 | M | 2 | 5.42 | 91.5 | c-B-ALL | / | / | Remission |
| 177 | F | 3 | 9.93 | 63 | c-B-ALL | / | / | Remission |
| 178 | F | 10 | 3.93 | 99 | c-B-ALL | / | / | Remission |
| 179 | M | 12 | 9.43 | 88.5 | c-B-ALL | / | / | Remission |
| 180 | M | 5 | 12.41 | 90 | c-B-ALL | / | / | Remission |
| 181 | M | 12 | 14.96 | 97 | c-B-ALL | / | / | Remission |
| 182 | M | 3 | 14.79 | 92.5 | c-B-ALL | / | / | Remission |
| 183 | M | 2 | 3.85 | 93.5 | c-B-ALL | / | / | Remission |
| 184 | M | 5 | 9.61 | 94.5 | c-B-ALL | / | / | Remission |
| 185 | F | 12 | 2.92 | 91 | c-B-ALL | / | / | Remission |
| 186 | M | 2 | 9.93 | 89 | c-B-ALL | / | / | Remission |
| 187 | M | 0.92 | 61.15 | 84 | pro-B-ALL | / | / | Remission |
| 188 | M | 6 | 16.97 | 39.5 | pro-B-ALL | / | / | Relapse |
| 189 | F | 6 | 22.66 | 97 | pre-B-ALL | / | / | Remission |
| 190 | M | 3 | 3.91 | 99.2 | c-B-ALL | / | / | Remission |
| 191 | M | 6 | 10.14 | 80 | pro-B-ALL | / | / | Remission |
| 192 | M | 9 | 1.78 | 97.5 | c-B-ALL | / | / | Remission |
| 193 | F | 3 | 15.79 | 93 | c-B-ALL | / | / | Remission |
| 194 | M | 7 | 55.1 | 97 | c-B-ALL | / | / | Remission |
| 195 | F | 9 | 4.17 | 99 | pro-B-ALL | / | / | Remission |
| 196 | F | 9 | 4.97 | 95 | c-B-ALL | / | / | Remission |
| 197 | F | 9 | 94.99 | 95 | c-B-ALL | / | / | Remission |
| 198 | F | 1 | 2.48 | 96 | c-B-ALL | / | / | Remission |
| 199 | F | 5 | 18.07 | 93.5 | c-B-ALL | / | / | Remission |
| 200 | F | 2 | 12.56 | 88.5 | c-B-ALL | / | / | Remission |
| 201 | M | 1 | 102.51 | 96.5 | c-B-ALL | / | / | Remission |
| 202 | M | 13 | 105 | 94 | pro-B-ALL | / | / | Relapse |
| 203 | M | 11 | 6.48 | 90 | c-B-ALL | / | / | Remission |
| 204 | M | 3 | 2 | 93 | c-B-ALL | / | / | Remission |
| 205 | M | 3 | 5.33 | 80 | c-B-ALL | / | / | Remission |
| 206 | M | 7 | 5.04 | 66.5 | c-B-ALL | / | / | Remission |
| 207 | M | 1 | 3.61 | 85 | c-B-ALL | / | / | Remission |
| 208 | M | 1 | 111.98 | 95.5 | c-B-ALL | / | / | Relapse |
| 209 | F | 4 | 4.08 | 50.5 | c-B-ALL | / | / | Remission |
| 210 | M | 8 | 8.28 | 84 | c-B-ALL | / | / | Remission |
| 211 | M | 4 | 4.92 | 95 | c-B-ALL | / | / | Remission |
| 212 | M | 1 | 199.5 | 95.5 | c-B-ALL | / | / | Relapse |
| 213 | M | 4 | 5.3 | 48.5 | c-B-ALL | / | / | Remission |
| 214 | M | 5 | 4.54 | 85.5 | c-B-ALL | / | / | Remission |
| 215 | M | 4 | 50.37 | 95 | c-B-ALL | / | / | Remission |
| 216 | M | 12 | 96.21 | 75 | pro-B-ALL | / | / | Relapse |
| 217 | F | 3 | 26.14 | 94.5 | c-B-ALL | / | / | Remission |
| 218 | F | 4 | 1.1 | 97.5 | c-B-ALL | / | / | Remission |
| 219 | M | 2 | 12.77 | 92 | c-B-ALL | / | / | Remission |

WBC, white blood cell; PB, peripheral blood; ID, initial diagnosis; BM, bone marrow; T-ALL, T lineage ALL; c-B-ALL, common B lineage ALL; pre-B-ALL, precursor B lineage ALL; pro-B-ALL, progenitor B lineage ALL.

**Table S2**

**Primers of the marker genes in the improved AFA multiplex assay**

| **RT-PCR Set** | **Gene ID** | **Gene Symbol** | **Forward primer** | **Reverse primer** | **RT Primer Concentration (nM)** | **PCR Primer Concentration (nM)** |
| --- | --- | --- | --- | --- | --- | --- |
| Set 1 | 8842 | PROM1 | TCTCCCTGTTGGTGATTTGT | CCAGTTTCCGACTCCTTTTG | 75.3 | 66.7 |
|  | 567 | B2M | CCGTGTGAACCATGTGACTT | ATTCATCCAATCCAAATGCGG | 26.6 | 66.7 |
|  | 4778 | NFE2 | GAACTGACTTGGCAGGAGAT | GTGGTGGAGGTCCAAGGTAT | 163 | 66.7 |
|  | 7535 | ZAP70 | CGCTGCACAAGTTCCTGG | CGGTGCACAAAGTTCTTCTC | 333.3 | 66.7 |
|  | 23089 | PEG10 | CTGAGACTCCATTTTGCTGC | CTCCTCTTCATGTCAGGCAA | 157.7 | 66.7 |
|  | 11119 | BTN3A1 | GAGCACAATGAAGCAAGAACA | AAGAGGGCCTTTTTCCATTCA | 215.7 | 66.7 |
|  | 1604 | DAF | GTGAAAATTCCTGGCGAGAAG | ATAAGGCTGTTTGAGGGATGC | 130 | 66.7 |
|  | 22862 | FNDC3A | ACGTGCAGCTAACAAAATGG | AAGGAACCTCCCAATTCACT | 109 | 66.7 |
|  | 10643 | IMP-3 | TCCACCGTAAAGAAAATGCG | TCAAGGGGATCTCTTCTGTG | 157 | 66.7 |
|  | 4192 | MDK | CAAAGGCCAAAGCCAAGAAA | AGCTAACGAGCAGACAGAAG | 333.3 | 66.7 |
|  | 969 | CD69 | CTCTTTGCATCCGGAGAGTG | GCCCACTGATAAGGCAATGA | 54.3 | 66.7 |
|  | 87 | ACTN1 | AAATCCAGACCCTAGCACG | GTGGGAGTTACACCATGCC | 108 | 66.7 |
|  | 3482 | IGF2R | GACGGCTGCAATCAATGAAA | CCTGCTCTCTATGATGCACT | 157.3 | 66.7 |
|  | 7102 | TSPAN7 | CAGTTAATTGGCATGCTGCT | ATGTACTGTGCTCTAACATTCT | 232 | 66.7 |
|  | 4068 | SH2D1A | GGCAAAATCAGCAGGGAAAC | CTCAGCACTCCAAGAACCTG | 163 | 66.7 |
|  | 50649 | ARHGEF4 | AGGAGGAAAAGTGAACCGAC | CTTCTCACCTGCTGGTTCTT | 333.3 | 66.7 |
|  | 1293 | COL6A3 | CGTCCAACAGGTCATCTCTG | GTCAACCAGCCTCTCTATGA | 1000 | 88.9 |
|  | 6782 | STCH | ATTGAAGCTGCTAACCTTGC | ATTGTTTCCAGACATTGCTCG | 333.3 | 66.7 |
|  | 5704 | PSMC4 | GGAAGACCATGTTGGCAAAG | TGAGCATCGAATCTCTTGGT | 158.7 | 66.7 |
|  | 26112 | DKFZP434C171 | AGGAGTCCATTCTGAGCCGA | TTCCTGTTGCAGGGTCGTAA | 229.3 | 66.7 |
|  | 2791 | GNG11 | AGTGAAGTTGCAGAGACAACA | AGCATGTTGGTTTTATCTGGG | 1000 | 88.9 |
|  | 2990 | GUSB | GCTTCGAGGAGCAGTGGTA | TTCACCCACACGATGGCATA | 333.3 | 66.7 |
|  | - | pcDNA 3.1 (+) | CAGACAATCGGCTGCTCTGA | GCTTCAGTGACAACGTCGA | 66.7 | 66.7 |
|  | - | KanR | CGGGAAAACAGCATTCCAGG | GTGACGACTGAATCCGGTGA | 333 | 66.7 |

| **RT-PCR Set** | **Gene ID** | **Gene Symbol** | **Forward primer** | **Reverse primer** | **RT Primer Concentration (nM)** | **PCR Primer Concentration (nM)** |
| --- | --- | --- | --- | --- | --- | --- |
| Set 2 | 4211 | MEIS1 | TTTTCACACTGGCCTTAAAGAG | GCCCCCGTAATGGGGTA | 12 | 66.7 |
|  | 567 | B2M | CCGTGTGAACCATGTGACTT | ATTCATCCAATCCAAATGCGG | 0.95 | 66.7 |
|  | 7738 | ZNF184 | TCGGCTCCCTGCGTTA | AACACGCTGAGGTTGTCTAC | 50 | 66.7 |
|  | 22795 | NID2 | TTTCACAGCCCACATCTCTC | GACCATGTTTGGTTGATTGC | 166.7 | 66.7 |
|  | 29760 | BLNK | ACGTGACCACTGGACAGTTA | AATATCATGGACCATCTTTTGAAGC | 33.3 | 66.7 |
|  | 5087 | PBX1 | ACTCAGTGGAGCATTCAGAT | CTCTCGCAGGAGATTCATCA | 65.3 | 66.7 |
|  | 5796 | PTPRK | CCCATCCAAGTGGAATGTATG | AAGCCCATCCTAGGTACTGA | 5.5 | 66.7 |
|  | 7133 | TNFRSF1B | ATGCCGGCTCAGAGAATAC | AGAGCTGGGTGTATGTGCT | 17 | 66.7 |
|  | 1687 | DFNA5 | CCTACTTCTTGGTCAGTGCC | GTTGGGTCTTCAAGATCAGATAC | 100 | 66.7 |
|  | 9467 | SH3BP5 | GGAGAAGAAACTCAAGAGAGC | TTCAGGGCCATCTTGTACTC | 14.8 | 66.7 |
|  | 55556 | ENOSF1 | GGCAGTGTCAATGAGAACCT | CTCACACACCCTATTTTCAAGG | 333.3 | 66.7 |
|  | 9590 | AKAP12 | GATAAAGCGATCACACCCCA | CAGCAGCAGCATTCATTTTC | 19.7 | 66.7 |
|  | 3655 | ITGA6 | ACGCGGATCGAGTTTGATAA | CAAAGATGTCTCGGGATTCCT | 12.9 | 66.7 |
|  | 10602 | CDC42EP3 | CAGTAAGCCACCCACTAGACA | AGCCAGTACCAATAAGTTAACGTC | 16.7 | 66.7 |
|  | 5704 | PSMC4 | GGAAGACCATGTTGGCAAAG | TGAGCATCGAATCTCTTGGT | 6.67 | 66.7 |
|  | 51465 | UBE2J1 | AGATCATTACCATGCGCAGC | CTTGCCCACTTCAAATCGAC | 5.7 | 66.7 |
|  | 8503 | PIK3R3 | CGCAGAGAGGGGAATGAAAA | GATCTTTCGCAGCTGGATCA | 100 | 66.7 |
|  | 23150 | FRMD4B | GTCTTCTACAAATGCTTCTGGG | GTCTCTGCTCACTACTCTCC | 59.3 | 66.7 |
|  | 11027 | LILRA2 | AGCCACAATCACTCATCAGAGTA | GGGCATGGGAATGGGAGTTC | 66.7 | 66.7 |
|  | 1362 | CPD | ACCGGATATTTGGTTTGCCA | GGAACTCATGGCTTAGGAGG | 42 | 66.7 |
|  | 2990 | GUSB | GCTTCGAGGAGCAGTGGTA | TTCACCCACACGATGGCATA | 73.3 | 66.7 |
|  | - | pcDNA 3.1 (+) | CAGACAATCGGCTGCTCTGA | GCTTCAGTGACAACGTCGA | 66.7 | 66.7 |
|  | - | KanR | CGGGAAAACAGCATTCCAGG | GTGACGACTGAATCCGGTGA | 167 | 57.8 |

| **RT-PCR Set** | **Gene ID** | **Gene Symbol** | **Forward primer** | **Reverse primer** | **RT Primer Concentration (nM)** | **PCR Primer Concentration (nM)** |
| --- | --- | --- | --- | --- | --- | --- |
| Set 3 | 7249 | TSC2 | GACATCATCATCAAGGCGCT | AAAGTTCCTGTAGAGGTGCG | 50.3 | 66.7 |
|  | 567 | B2M | CCGTGTGAACCATGTGACTT | ATTCATCCAATCCAAATGCGG | 20.5 | 66.7 |
|  | 2770 | GNAI1 | CAGAACTAGAGTGAAAACTACAGG | TGAATCCACTTCTTCCGCTC | 166.7 | 66.7 |
|  | 5101 | PCDH9 | GGCAACTCTGATCCCAACTC | CAGGAGGCATCCAGCAATTA | 112 | 66.7 |
|  | 240 | ALOX5 | TGCACATGTTCCAGTCTTCT | ACTGGTAGCCAAACATCAGG | 31.9 | 66.7 |
|  | 150094 | SNF1LK | GAAGCTTCTGAACCATCCAC | CTCGTTCTCACTCAGGTGC | 154.7 | 66.7 |
|  | 2195 | FAT | CCCCTTTGGTTTGACATCAC | GTTCTGCATCAAGAGGTTTGG | 84 | 66.7 |
|  | 1490 | CTGF | GTTACCAATGACAACGCCTC | GCTCAAACTTGATAGGCTTGG | 23.3 | 66.7 |
|  | 663 | BNIP2 | AATTTGGCAGAACTAGCAGA | CACATTCTTCAGTCTTGTTTGGA | 87.3 | 66.7 |
|  | 9452 | ITM2A | TCTGATGCCCCTCAATACTTC | AGATGCCAAGGTTACTAACATCA | 280 | 66.7 |
|  | 3385 | ICAM3 | TGACTGGCAACAGTCGGAT | TCCACTTGGCAGCGCA | 166.7 | 66.7 |
|  | 4082 | MARCKS | GTTTCCCCTCTTGGATCTGT | TACCTTCACGTGGCCATTC | 166.7 | 66.7 |
|  | 9934 | P2RY14 | CTTCACTGAAAAGAGACCTCA | GCAATGAAGACCATACAGTACA | 166.7 | 66.7 |
|  | 5366 | PMAIP1 | GCTCTGTAGCTGAGTGGG | AGAAGAGTTTGGATATCAGATTCAG | 166.7 | 66.7 |
|  | 5937 | RBMS1 | GTGTTGGCTTTGCTAGGATG | ATGCCATGGTCTTCCATTAGG | 166.7 | 66.7 |
|  | 27245 | AHDC1 | GTGTTGGCTTTGCTAGGATG | ATGCCATGGTCTTCCATTAGG | 166.7 | 66.7 |
|  | 5704 | PSMC4 | GTGTTGGCTTTGCTAGGATG | ATGCCATGGTCTTCCATTAGG | 92.3 | 66.7 |
|  | 8936 | WASF1 | AAAGAGGAAGCAGAAGCAGA | GATCCACGTATGTCTGAGGT | 113.3 | 66.7 |
|  | 219654 | C10 or F56 | CAGCTATCTCAACAGCTTCTTC | TTTGTTGAAGCACAGGTGGC | 333.3 | 66.7 |
|  | 53405 | CLIC5 | AAAAATTAAACACCGCCCTGA | ACAGTTGCCGATGCTTTCT | 166.7 | 66.7 |
|  | 3512 | IGJ | AACCATTTGCTTTTCTGGGG | TGAGGTGGGATCAGAGATATT | 288.3 | 66.7 |
|  | 2990 | GUSB | GCTTCGAGGAGCAGTGGTA | TTCACCCACACGATGGCATA | 496 | 66.7 |
|  | - | pcDNA 3.1 (+) | CAGACAATCGGCTGCTCTGA | GCTTCAGTGACAACGTCGA | 66.7 | 66.7 |
|  | - | KanR | CGGGAAAACAGCATTCCAGG | GTGACGACTGAATCCGGTGA | 100 | 66.7 |

**Table S3**

**Prediction results for 219 iAFA samples**

| Subtype | TP | FP | TN | FN | Accuracy | Sensitivity | Specificity |
| --- | --- | --- | --- | --- | --- | --- | --- |
| *BCR-ABL1* | 4 | 17 | 193 | 5 | 89.95% | 44.44% | 91.90% |
| *E2A-PBX1* | 12 | 0 | 207 | 0 | 100.00% | 100.00% | 100.00% |
| Hyperdiploid | 61 | 35 | 113 | 10 | 79.45% | 85.92% | 76.35% |
| *MLL* rearrangement | 1 | 1 | 213 | 4 | 97.72% | 20.00% | 99.53% |
| Others | 1 | 0 | 169 | 49 | 77.63% | 2.00% | 100.00% |
| T-ALL | 16 | 2 | 201 | 0 | 99.09% | 100.00% | 99.01% |
| *TEL-AML1* | 56 | 13 | 150 | 0 | 94.06% | 100.00% | 92.02% |

TP: True Positive; FP: False Positive; TN: True Negative; FN: False Negative.

Accuracy= (TP+ TN)/(TP+ FN+ TN+ FP); Sensitivity= TP/(TP+ FN); Specificity= TN/(TN+ FP).

Average accuracy: 91.13%.

**Table S4**

**Parameter estimation of the optimal prediction model**

| **Term** | **Estimate** | **Std Error** | **χ^2^** | ***P*** |
| --- | --- | --- | --- | --- |
| Intercept | -2.6431971 | 0.2643924 | 99.95 | <.0001 |
| *ITM2A* | 0.49080283 | 0.2015175 | 5.93 | 0.0149 |
| *COL6A3* | 0.35062101 | 0.1448491 | 5.86 | 0.0155 |
| *PTPRK* | -1.0370042 | 0.3620418 | 8.20 | 0.0042 |
| *RBMS1* | 0.52098988 | 0.1571118 | 11.00 | 0.0009 |
| *IMP-3* | 0.46767229 | 0.1871628 | 6.24 | 0.0125 |
| *ALOX5* | -0.5576611 | 0.2357152 | 5.60 | 0.0180 |
| *CD69* | 0.41004192 | 0.1586229 | 6.68 | 0.0097 |
| *STCH* | -0.6606856 | 0.331085 | 3.98 | 0.0460 |
| *FNDC3A* | -0.42502 | 0.2317615 | 3.36 | 0.0667 |
| *PROM1* | 0.2921352 | 0.1505781 | 3.76 | 0.0524 |

**Table S5**

**Twenty-seven children in the previous 160 ALL cases with dismal prognosis**

| **NO.** | **Sex** | **Age of diagnosis (years)** | **WBC in PB at ID (×10^9^/L)** | **Percentage of blast cells in BM at ID (%)** | **Immunotype** | **Cytogenetic abnormality** | **Fusion gene** | **Outcome** |
| --- | --- | --- | --- | --- | --- | --- | --- | --- |
| 1 | M | 7 | 144.8 | 86 | T-ALL | － | － | Relapse |
| 3 | M | 5 | 70.5 | 83 | c-B-ALL | t(1;19)(q23;p13) | *E2A-PBX1* | Relapse |
| 4 | M | 8 | 59.1 | 72 | T-ALL | － | － | Death |
| 7 | M | 9 | 9.5 | 77.5 | c-B-ALL | t(9;22)(q34;q11) | *BCR-ABL1* | Relapse |
| 9 | M | 5 | 142.9 | 89.5 | c-B-ALL | t(9;22)(q34;q11) | *BCR-ABL1* | Death |
| 11 | M | 4 | 22.2 | 91 | c-B-ALL | t(1;19)(q23;p13) | *E2A-PBX1* | Death |
| 12 | M | 4 | 38 | 90 | c-B-ALL | t(9;22)(q34;q11) | *BCR-ABL1* | Death |
| 17 | M | 5 | 378.2 | 84 | c-B-ALL | t(9;22)(q34;q11) | *BCR-ABL1* | Relapse |
| 24 | F | 9 | 180 | 87 | T-ALL | － | － | Relapse |
| 30 | M | 10 | 61.7 | 87 | T-ALL | － | － | Death |
| 31 | M | 4 | 13.8 | 81 | c-B-ALL | t(1;19)(q23;p13) | *E2A-PBX1* | Relapse |
| 39 | M | 8 | 85.8 | 90 | T-ALL | － | － | Relapse |
| 40 | F | 3 | 6.38 | 71 | c-B-ALL | t(4;11)(q21;q23) | *MLL-AF4* | Death |
| 47 | M | 9 | 87.53 | 91 | c-B-ALL | t(9;22)(q34;q11) | *BCR-ABL1* | Relapse |
| 48 | M | 9 | 79.24 | 90 | c-B-ALL | t(9;22)(q34;q11) | *BCR-ABL1* | Death |
| 49 | M | 5 | 19.34 | 74 | c-B-ALL | t(12;21)(p13;q22) | *TEL-AML1* | Relapse |
| 55 | F | 5 | 284.87 | 93 | T-ALL | － | － | Death |
| 56 | F | 8 | 30.02 | 76 | c-B-ALL | t(9;22)(q34;q11) | *BCR-ABL1* | Death |
| 58 | M | 10 | 249.61 | 85 | c-B-ALL | t(9;22)(q34;q11) | *BCR-ABL1* | Death |
| 63 | M | 11 | 148.47 | 93 | T-ALL | del(1)(p34p34) | *SIL-TAL1* | Relapse |
| 69 | M | 2 | 9.13 | 90 | c-B-ALL | Hyperdiploid | - | Relapse |
| 75 | M | 12 | 98.17 | 90 | T-ALL | del(1)(p34p34) | *SIL-TAL1* | Relapse |
| 111 | F | 3 | 3.78 | 80 | c-B-ALL | Hyperdiploid | *－* | Death |
| 116 | M | 5 | 3.66 | 85 | c-B-ALL | Hyperdiploid | *－* | Relapse |
| 126 | M | 8 | 207.21 | 87 | T-ALL | del(1)(p34p34) | *SIL-TAL1* | Relapse |
| 133 | F | 5 | 37.4 | 95 | c-B-ALL | Hyperdiploid | *－* | Relapse |
| 157 | M | 11 | 5.58 | 81 | c-B-ALL | t(9;22)(q34;q11) | *BCR-ABL1* | Death |
